# MkVsites: A tool for creating GROMACS virtual sites parameters to increase performance in all‐atom molecular dynamics simulations

**DOI:** 10.1002/jcc.26198

**Published:** 2020-04-13

**Authors:** Per Larsson, Rosita C. Kneiszl, Erik G. Marklund

**Affiliations:** ^1^ Department of Pharmacy, Uppsala Biomedical Research Centre Uppsala University Uppsala Sweden; ^2^ Department of Chemistry – BMC, Uppsala Biomedical Research Centre Uppsala University Uppsala Sweden

**Keywords:** GROMACS, molecular dynamics, sampling, timestep

## Abstract

The absolute performance of any all‐atom molecular dynamics simulation is typically limited by the length of the individual timesteps taken when integrating the equations of motion. In the GROMACS simulation software, it has for a long time been possible to use so‐called virtual sites to increase the length of the timestep, resulting in a large gain of simulation efficiency. Up until now, support for this approach has in practice been limited to the standard 20 amino acids however, shrinking the applicability domain of virtual sites. MkVsites is a set of python tools which provides a convenient way to obtain all parameters necessary to use virtual sites for virtually any molecules in a simulation. Required as input to MkVsites is the molecular topology of the molecule(s) in question, along with a specification of where to find the parent force field. As such, MkVsites can be a very valuable tool suite for anyone who is routinely using GROMACS for the simulation of molecular systems.

## Graphical abstract

Insufficient sampling is a common problem for molecular dynamics simulations of biomolecules. With the virtual sites approach, all hydrogen atoms are maintained, but at the same time, mass‐less interaction sites are introduced. This means that the length of the timestep in a simulation can be increased by a factor of 2.5, giving a considerable performance boost practically for free. MkVsites is a python tool for obtaining virtual sites parameters for virtually any molecule in a simulation.

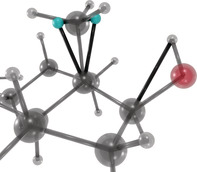

## 1 INTRODUCTION

For dynamic heterogeneous systems such as biological membranes, aggregation processes, and studies of protein folding and motion, molecular dynamics (MD) simulations enables the generation of mechanistic hypotheses via detailed physical models for observed phenomena. These advances are due to progress in parallel and stream computing, new molecular interaction parameters with increased physical accuracy, and development of methods that enable robust statistical estimates of physical observables.^[^ ^1^ ^]^ Ultimately, however, the amount of useful data that can be obtained from MD will always be limited by how many timesteps that can be afforded, and the length of those timesteps.

In atomistic simulations, the length of the timestep is limited by the fastest degrees of freedom in a molecule, typically bond vibrations in bonds involving hydrogen atoms.^[^ ^2^ ^]^ To get around this issue and increase the overall performance, a number of options have been described in the literature, including the use of united‐atom force fields,^[^ ^3^ ^]^ removing all but the polar hydrogen atoms, or putting explicit constraints on the bonds involving hydrogens.^[^ 4, 5 ^]^ These strategies still limit the timestep to 2 fs however. A number of studies seek to go beyond this by repartitioning the masses of hydrogen and connecting heavy atoms, making the hydrogens heavier while conserving overall mass of each molecule.^[^ 6, 7 ^]^ While such repartitioning will not perturb the free energy surface, it will inevitably affect dynamic properties.

An alternative means to increase the timestep is so‐called virtual interaction sites, which are used in conjunction with bond constraints. Here, the masses of the hydrogens together with all forces acting on the latter are redistributed to the adjacent heavy atoms, and integration of the equations of motion are then done based on the heavy atoms.^[^ ^2^ ^]^ After the positions of the heavy atoms have been updated during an integration step, the massless hydrogen positions get determined through linear or nonlinear combinations of the positions of the heavy atoms located one or two bonds away. For most aliphatic hydrogens, this operation is relatively straightforward and GROMACS can create the necessary parameters for virtually any new residue or ligand without additional information.

**Figure 1:**
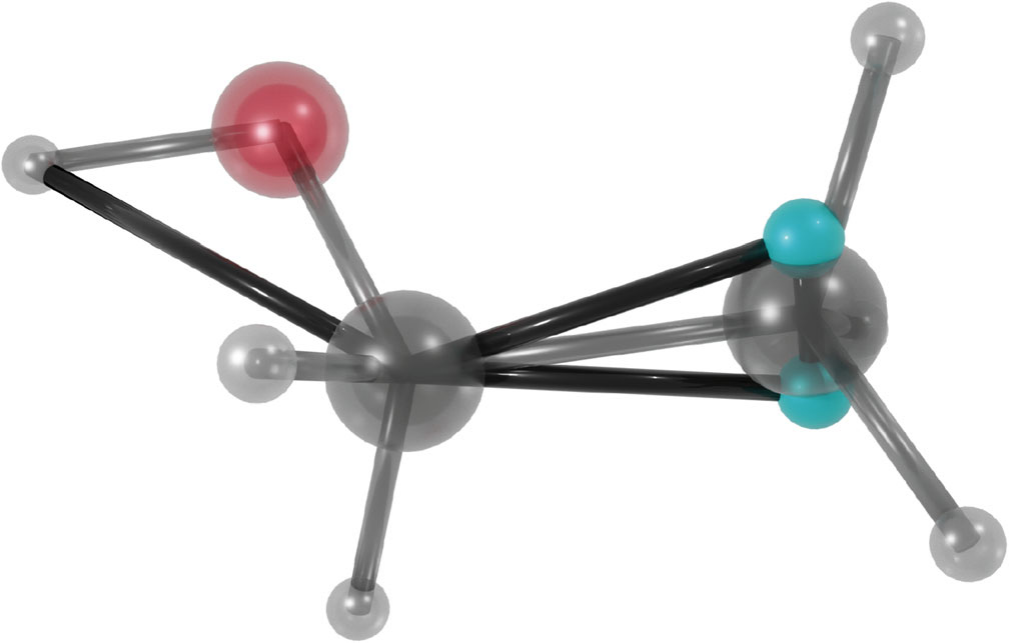
The image shows ethanol with a constraint connecting the hydoxyl hydrogen to the “anchor,” keeping the angle fixed, and two dummy particles (teal) for the methyl group. Virtual‐site specific constraints are shown as black rods. Note the triangle of constraints connecting the dummy particles and the anchor, keeping the center of mass and moment of inertia constant and correct [Color figure can be viewed at wileyonlinelibrary.com]

For some functional groups, the situation is more complicated, and more complex constructions that require additional parameters are needed. For example, –CH_3_ and –NH_3_ are able to spin around their axis, so that the hydrogen positions are not fully defined by the bonded heavy atoms. A solution is to add two dummy particles and let the combined mass of the group be completely redistributed to them, then let the hydrogens’ positions be determined from the dummy particles, analogous to the case with aliphatic hydrogens. Additionally, for groups like hydroxyls, which have a heavy atom connecting a single hydrogen to an “anchoring” heavy atom, a constraint can be added between the hydrogen and the anchor, keeping the angle constant while allowing free rotation of the group. These measures, illustrated in Figure 1, maintain the masses and moments of inertia of chemical groups as well as the interatomic interactions while disabling hydrogen vibrations. Consequently, the dynamics of the system are largely conserved, except for the fastest degrees of freedom, but in return, the timestep can be more than doubled to 5 fs for biomolecular systems.

Virtual site parameters are available in GROMACS^[^ 8, 9 ^]^ for simulations of the common amino acids, and more recently has also started to become available for lipids, and are widely used to increase the timestep.^[^ ^10^ ^]^ For most nonprotein molecules, however, no such parameters exist, effectively limiting the timestep and consequently, the sampling in all simulations including such components. Given the large number of biological relevant molecules, including drug molecules, carbohydrates, and post‐translationally modified proteins, this limitation is doubtlessly significant.

Virtual site parameters can be constructed from information in the force field and a molecular topology. Biomolecular force fields typically provide abstract description of the interatomic interactions via interaction parameters between types of atoms, where the atom type reflects not only the element, but also electronic properties such as hybridization, degree of polarization, and so on. The bonded interactions are normally expressed in terms of deviations from reference bond lengths, angles, dihedral angles, and so on. A topology file, on the other hand, provides a specific description of the interactions by stating which atoms are connected and which atom types they are. From the combination of these two descriptions, the ideal geometry of chemical groups, such as methyl and amines, can be determined, and their centers of mass and moments of inertia can be calculated in turn, which is all that is needed to make appropriate dummy‐mass constructions. Analogously, for hydroxyl‐like groups the ideal distance between the hydrogen and anchor can be precisely determined, defining the constraint length required to maintain their geometries.

Derivation of virtual site parameters currently requires a considerable manual effort in terms of finding bond parameters and atom type properties in force field file, as well as geometry calculations to determine constraint lengths and dummy‐mass positions. To greatly facilitate applying the virtual site approach for arbitrary biomolecules we here present MkVsites, a python tool that automates the calculations of all necessary parameters for any molecule such as ligands, noncanonical amino acids, lipids, nucleic acids, or small drug‐like compounds. MkVsites moreover provides additional functionality to facilitate the inclusion of newly parameterized molecules and residues in biomolecular simulation more generally.

## 2 METHODS

MkVsites works through two python programs: One that makes virtual site parameters for a given topology based on information in the force field (mkvsites.py), and one auxiliary program that extracts the interaction parameters that are not already in the force field (topinspect.py). Both take a molecular topology together with information from the force field and automate most of the process of complementing the force field with the required virtual site parameters.

### 2.1 Catching new atom types and interactions in the topology: topinspect.py

**Figure 2:**
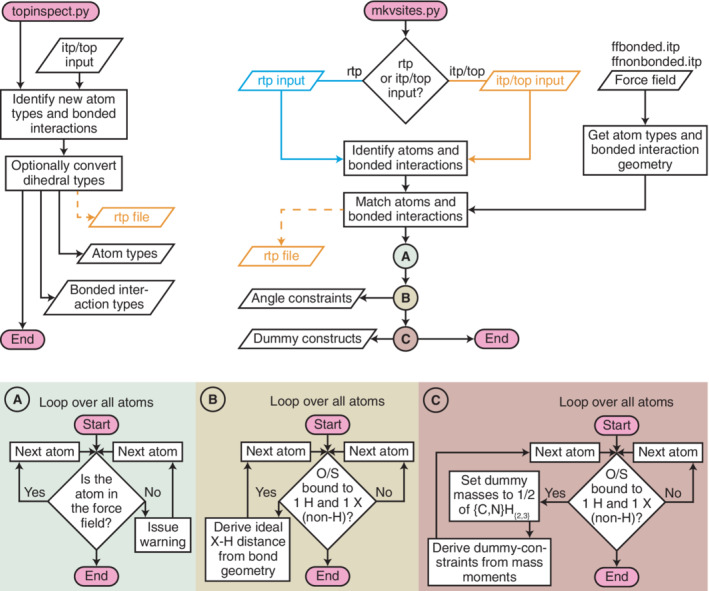
Simplified flowcharts describing the workings of MkVsites's two programs: topinspect.py and mkvsites.py. topinspect.py lists new atom types and interactions from a topology file, optionally converting the dihedrals from one type to another, and optionally creating an rtp file for use with pdb2gmx. mkvsites.py reads a topology from an rtp or itp/top file and combines it with information from the force field to establish the geometry and the mass moments of chemical groups. After a check to ensure all atom types are found in the force field, the angle constraints for –OH and –SH are calculated, and new dummy particle atom types and the associated constraints and parameters are created [Color figure can be viewed at wileyonlinelibrary.com]

topinspect.py is an auxiliary program that is useful when a compound contains new atom types or have special interactions in its topology. For example, when having parameterized a new ligand. It is however not required for many biomolecular building blocks, such as nucleotides, that are already defined by the force field. Taking an itp/top file as input, topinspect.py identifies atom types and interactions that are not already present in the force field files and displays them in a manner that makes for an easy incorporation in the force field (Figure 2). While the latter step—incorporation into existing force field files—could in principle be automated, we have noted that some topology files contain ambiguous information about the elements of new atom types, which either require assumptions from the software or a manual step carried out by the user. We have chosen the latter for transparency reasons and for reducing the risk of silent errors entering the simulations. With the new atom types and interaction parameters incorporated, all the abstract information that is required for the generation of virtual sites parameters are now present in the force field.

topinspect.py provides additional functionality to facilitate the inclusion of ligands and new residues. First, it can use the input topology to optionally output a residue topology (rtp), which enables the GROMACS program pdb2gmx to go directly from a pdb file to a complete topology. This feature is handy when preparing a system for simulation in general, but it also makes use of the capability of pdb2gmx to generate all virtual sites constructions, including placing all dummy‐mass atoms exactly where they should be relative to other atoms and adding all associated constraints. Additionally, topinspect.py can optionally convert dihedrals from one functional form to another, which can be used to harmonize, for example, a newly parameterized noncanonical amino acid with existing amino acids in the force field. For example, the parametrization might yield dihedrals of a specific type, while existing rtp entries in the force field use another type. Specifically, topinspect.py converts between ordinary proper dihedrals, Fourier dihedrals, and Ryckaert–Belleman dihedrals. Conversion is impossible for some combinations of parameter values, due to their mathematical functional forms, in which case an error will be raised by the program. Moreover, conversion will most often yield a constant offset to the energy, which is normally not a problem as long as the gradient, and hence the forces in the simulation, is preserved. These two features—rtp creation from top/itp files, and conversion of dihedral parameters—have, to the best of our knowledge, not been implemented anywhere else, and make a time consuming and error‐prone manual operation fully automatic.

### 2.2 Automatic derivation of the virtual site parameters: mkvsites.py

mkvsites.py reads an itp/top or rtp file, and the force field files, to generate all parameters needed for applying virtual sites (Figure 2). It reads the input and searches for heavy atoms (nonhydrogen) with only one other heavy‐atom bound. If the atom is also bound to three hydrogen atoms (–CH_3_ and –NH_3_ groups) the mass moments are calculated for making dummy particles, for which new atom types and necessary default constraints are generated (Figure 1). If only one hydrogen is bound to the heavy atom (currently only –OH and –SH), then a constraint between the bound heavy atom and the hydrogen is generated to keep the angle rigid. mkvsites.py derives all parameters from the data already present in the force field (after potential additions from topinspect.py). The new parameters and dummy atoms types are printed to the terminal for incorporation in the force field files. MkVsites does not generate parameters for, for example, aliphatic hydrogens, since they are handled automatically by GROMACS without need of additional parameters.

### 2.3 Testing and validating MkVsites

To test the accuracy of the dihedral conversion implemented in topinspect.py, we created a four‐atom molecule without non‐bonded interactions, where the atoms were connected in sequence with angles of 109.5°. The molecule thus had one dihedral angle, which we rotated one full revolution in steps of 10°. Three different topologies were created, based on an original Ryckaert–Belleman dihedral potential, and the conversion to Fourier and proper dihedrals. The topologies and the “trajectory” of the dihedral scan was used as input to GROMACS to calculate the energies.

To validate and showcase the usefulness of MkVsites, simulations with and without virtual sites on the protein and ligand were started from the crystal structure of the estrogen receptor, PDB ID 5TOA, using the amber99sb^[^ ^11^ ^]^ force field. Some missing atoms were first added using Modeler,^[^ ^12^ ^]^ and a topology (initially without virtual sites) for the estrogen cofactor was generated using the Stage^[^ ^13^ ^]^ software with the GAFF force field. An rtp‐file was created from the topology file to allow us to use the existing virtual‐sites machinery in the GROMACS tool pdb2gmx. Virtual site parameters for the bound cofactor was derived using MkVsites: ./mkvsites.py ‐ff /path/to/amber99sb.ff ‐r EST EST.rtp.

The initial structures were solvated in a box with roughly 29,000 TIP3P water molecules in each case. Long‐range electrostatics were calculated using PME^[^ ^14^ ^]^ using a real‐space cutoff of 1.0 nm. Lennard‐Jones interactions were evaluated using a straight 1.0 nm cutoff. Both systems were first energy minimized for 500 steps using steepest descent, and simulations were then run for 500 ns each using either a 2 or 5‐fs time step at a temperature of 310 K maintained by the V‐rescale thermostat^[^ ^15^ ^]^ and a pressure of 1 bar with the Parrinello–Rahman barostat.^[^ ^16^ ^]^ Hydrogen bond analysis as well as root‐mean‐square calculations were done using standard GROMACS tools, and molecular graphics generated with Blender.

## 3 RESULTS

**Figure 3:**
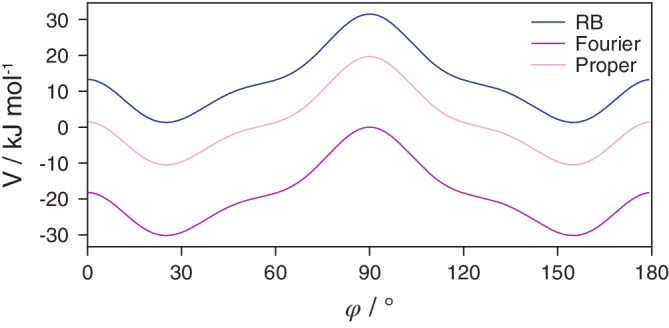
Energies calculated using GROMACS for the Ryckaert–Belleman, Fourier, and proper dihedrals. The parameters for latter two were obtained via topinspect.py by conversion from the Ryckaert–Belleman form. The energies have an expected constant offset, but have indistinguishable gradients, yielding identical forces in simulations [Color figure can be viewed at wileyonlinelibrary.com]

To ensure that conversion of dihedrals from one form to another does not affect the forces calculated during a simulation, we compared the energies from Ryckaert–Belleman dihedrals with Fourier and proper dihedrals converted from the former using GROMACS. Apart from an expected constant energy offset between the three dihedral types, the energy profiles were indistinguishable (Figure 3), validating the implementation.

**Figure 4:**
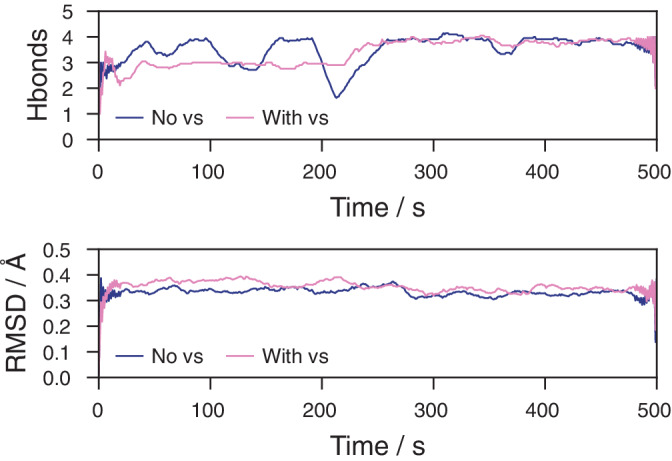
Simple quality measures for a set of simulations, performed for a complex with the estrogen receptor with bound estrogen, using both a 2 and 5 fs timestep. Neither the total number of hydrogen bonds between the ligand and the receptor (top) or the RMSD of the ligand (bottom) over the 500‐ns simulation period show significant differences. All lines show running averages using a window of 20‐ns width, except near the endpoints where the window is narrowed down symmetrically to fit within the data range, giving the appearance of locally larger fluctuations [Color figure can be viewed at wileyonlinelibrary.com]

**Figure 5:**
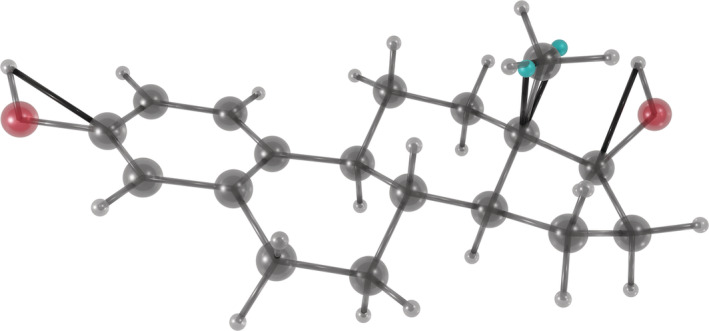
Snapshot of a single estrogen molecule with one methyl group having dummy masses, and with angle constraints for the two hydroxyl groups. All other hydrogen atoms are virtual interaction sites that are already handled by GROMACS automatically [Color figure can be viewed at wileyonlinelibrary.com]

To test and illustrate the usefulness of MkVsites, we performed two simulations of a hormone‐bound estrogen receptor (Figure 4). The estrogen molecules, parametrized through the GAFF force field, contains atom types which are not found in the parent amber99sb force field files (even though the atom types are commonly used in GAFF). This highlights one of the key strengths with MkVsites, that it works also in cases where a molecule previously not possible to represent with virtual sites also contain new atom types (Figure 5).

## 4 CONCLUSIONS

MkVsites provides the research community with a tool that makes it possible and simple to derive necessary virtual site parameters for any molecule. This means that the virtual sites‐approach for enhanced simulation performance can be applied for all simulations, as long as the corresponding molecular topologies can be derived in a manner consistent with the parent force field. While MkVsites was developed for GROMACS formats, the inner workings of the software are based on general concepts such as bonds, angles, and so on. As such, one can extend MkVsites with interfaces for other force‐field and topology formats to derive parameters for a wider range of simulation packages. Although the virtual sites method is currently only fully implemented in GROMACS (to our knowledge), MkVsites could be of utility for future implementations, and at least the angle constraints should be easy to use immediately. Moreover, MkVsites has been developed for simulations of biological or organic molecules, but can easily be extended to other classes of compounds that include other, heavier elements as long as their bound hydrogens can be handled using existing virtual sites models.

MkVsites is written in Python and requires no special external libraries. MkVsites can be downloaded and installed from https://github.com/ErikMarklund/mkvsites, where additional documentation and more usage examples can also be found.
